# Leukemia Cutis of the Face, Scalp, and Neck Treated with Non-coplanar Split Field Volumetric Modulated Arc Therapy: A Case Report

**DOI:** 10.7759/cureus.430

**Published:** 2015-12-23

**Authors:** Kyle Stang, Fiori Alite, Jennifer Steber, Bahman Emami, Murat Surucu

**Affiliations:** 1 Department of Radiation Oncology, Loyola University of Chicago Stritch School of Medicine; 2 Department of Radiation Oncology, Loyola University of Chicago Stritch School of Medicine

**Keywords:** leukemia cutis, radiation, volumetric modulated arc technique, vmat

## Abstract

Malignancies with a superficial involvement of the scalp present a unique technical challenge for radiation treatment planning. As an example of this, leukemic infiltration of the superficial skin as the only presentation of the disease is a rare entity. For such cases, radiation oncologists have typically treated with 3D conformal radiotherapy with matched electron fields, a technique that can lead to significant dose inhomogeneity. In this report, we describe the case of a patient with leukemia cutis with a superficial involvement of the scalp, face, and shoulders that was treated with volumetric modulated arc radiotherapy, with an impressive clinical response.

## Introduction

Malignancies with a superficial involvement of the scalp present a unique technical challenge for radiation treatment planning. Common malignancies treated with scalp irradiation include squamous and basal cell carcinoma, mycoses fungoides, melanoma, angiosarcoma, cutaneous lymphoma, and sarcoma [1]. The specific technical challenges inherent in the presentation of these tumors include their often multifocal spread, irregular borders on the convex surface of the calvarium, and a target volume which is in close proximity with the skin surface. Additionally, the resource burden of patient setup (i.e. movement restriction), staff-intensive treatment planning time, and linear accelerator burden also contribute to these complex treatment scenarios.

Techniques used in the past to address these challenges vary widely and include electron beam and photon beam techniques (either separate or combined), intensity-modulated radiation therapy (IMRT), and high-dose-rate brachytherapy, among others [2-4].

To our knowledge, a robust analysis of the most effective scalp irradiation technique for these cutaneous malignancies has not been undertaken. Indeed, the idiosyncrasies of a given tumor presentation, as well as factors limiting treatment planning such as planning time or equipment availability, often dictate the chosen technique. This report describes our institution’s experience with a novel radiation technique for a case of leukemia cutis involving the scalp and facial skin. 

## Case presentation

A 73-year-old male presented with a six-year history of relapsing/remitting diffuse erythematous facial and scalp rash. He had failed multiple rounds of antibiotic and steroid therapy. He was diagnosed initially with sensitivity to CL + Me-Isothiazolinone present in certain shampoos after allergen testing, but product avoidance did not improve his symptoms. Eventually, skin biopsy confirmed predominantly B cell infiltrate, and bone marrow biopsy demonstrated chronic lymphocytic leukemia/small lymphocytic leukemia (CLL/SLL) CD 38+, trisomy 12. The systemic staging was negative for any lymphoid involvement, confirming the diagnosis of leukemia cutis. The extent of superficial involvement of the malignancy included the scalp, face, and neck with thickened involvement primarily in the forehead and in a malar distribution. Due to the lack of clinical systemic involvement, he was recommended to undergo primary radiation therapy. The risks, benefits, and alternatives of radiation therapy in this context were discussed in detail with the patient, including acute and long-term side effects of radiation in this location. Total scalp electron beam therapy or systemic chemotherapy were discussed as alternative options, but photon volumetric arc therapy was pursued due to potential superior dose distribution. With cutaneous-only involvement, up front systemic chemotherapy was felt to be an inferior option. Informed patient consent was obtained prior to treatment.

**Figure 1:**
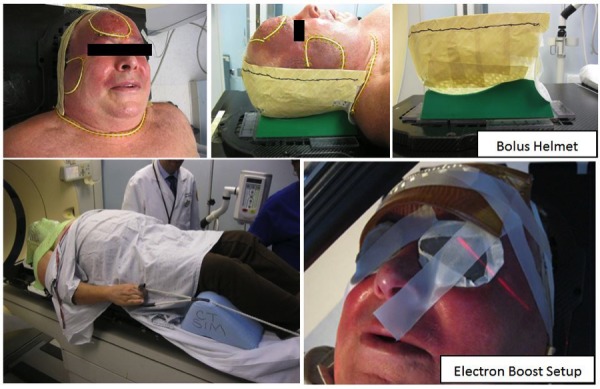
Simulation and Setup

The patient underwent a supine planning CT scan with the ‘C’ angle headrest. A custom 0.5 cm bolus was placed to encompass the entire face, scalp, cheeks, and shoulders. An aquaplast head and neck immobilization mask was fashioned with an overlying bolus helmet (Figure 1).

**Figure 2:**
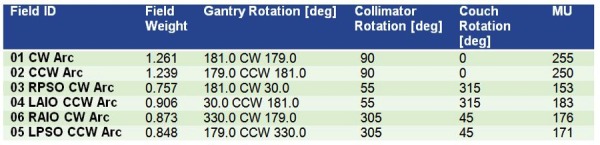
Treatment Planning - Fields Non-coplanar arc arrangement, collimator rotations selected to obtain optimal MLC leaf pattern (CW= Clockwise; CWW= Counterclockwise; RPSO= Right Posterior Superior Oblique; LAIO=Left Anterior Inferior Oblique; RAIO= Right Anterior Inferior Oblique; LPSO= Left Posterior Superior Oblique)

**Figure 3:**
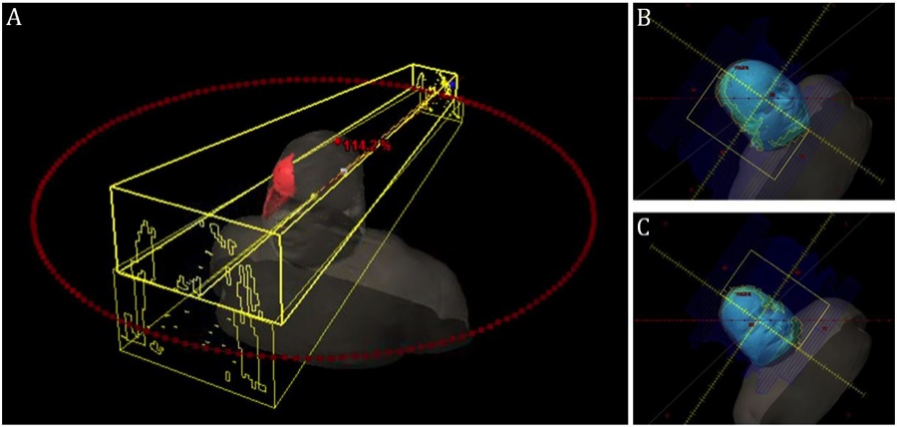
Treatment Planning - Beams A: Arc distribution of coplanar fields displaying 90 degree collimator rotations. B: Beams Eye View and MLC leaf shape of RPSO CW Arc. C: Beams Eye View and MLC leaf shape for LAIO CCW Arc

**Figure 4:**
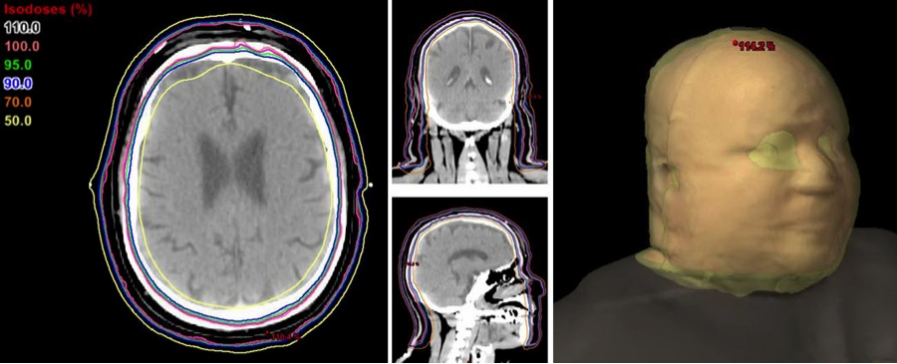
3D Isodose Distribution Isodose distribution in three cross-sectional planes as well as 3D dose cloud, highlighting dose distribution throughout total scalp, skin of face, cheeks, chin, neck, and upper shoulder

The inferior extent of the shoulder lesions was outlined with radiopaque wire, and an area of thickened bulky lesion in the forehead was outlined with radiopaque wire. The bulky skin lesions were delineated as gross tumor volume (GTV); the entire scalp, forehead, face, and inferiorly to the shoulder was delineated as clinical target volume (CTV). A uniform 3 mm margin was applied to CTV. The patient was treated with 20 Gy in 10 fractions, daily Monday to Friday, and 0.5 cm of bolus material was applied daily to the overlying skin. Six split-field, non-coplanar arcs were employed to optimize the plan and to obtain the dose distribution. The collimator settings for coplanar arcs were set at 90 degrees to better spare the brain, as previously described by Kelly, et al. [5]. See Figures 2-4 for detailed field arrangements and isodose distributions.

**Figure 5:**
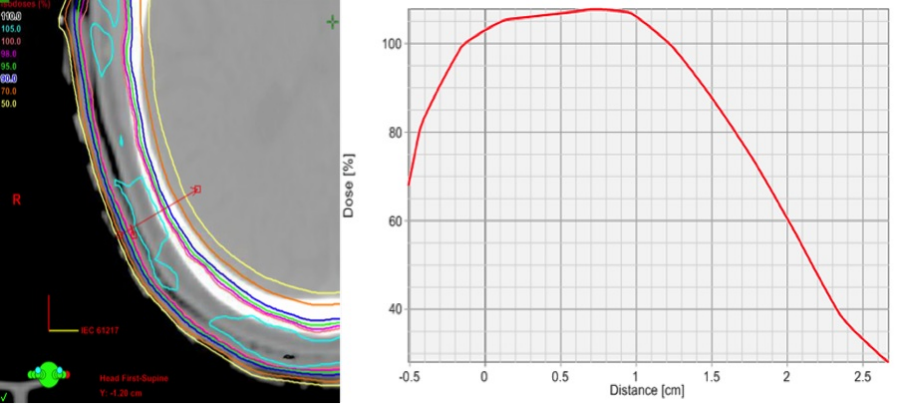
Depth Dose Characteristics Isodose line distribution at scalp surface and dose profile curve, highlighting depth dose characteristics comparable to electron dosimetry.

Figure 5 highlights depth dose characteristics of the IMRT plan from the surface of the scalp, which are comparable to electron dosimetry, and yet retain their characteristics throughout the different angles of the scalp. 

**Figure 6:**
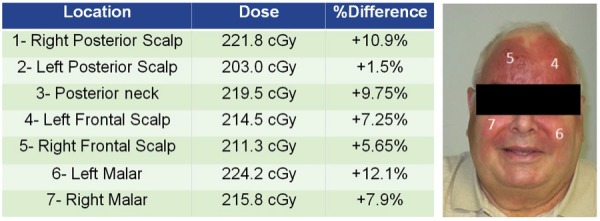
OSLD Measurements Seven OSLDs were placed as described to verify in vivo dose

The plan IMRT QA resulted in 98.6% average, 2D percentage of pixels passing Gamma analysis. Seven optical stimulated luminescence dosimeters (OSLDs) were placed as described to verify the in-vivo dose, which corresponded closely to the calculated dose (Figure 6).

The bulky forehead lesion was boosted with en-face electrons 10 Gy in five fractions, prescribed to the 90% IDL. An initial right malar bulky lesion responded during treatment, and the decision was made not to boost. Lead external eye shields were utilized during electron beam delivery.

**Figure 7:**
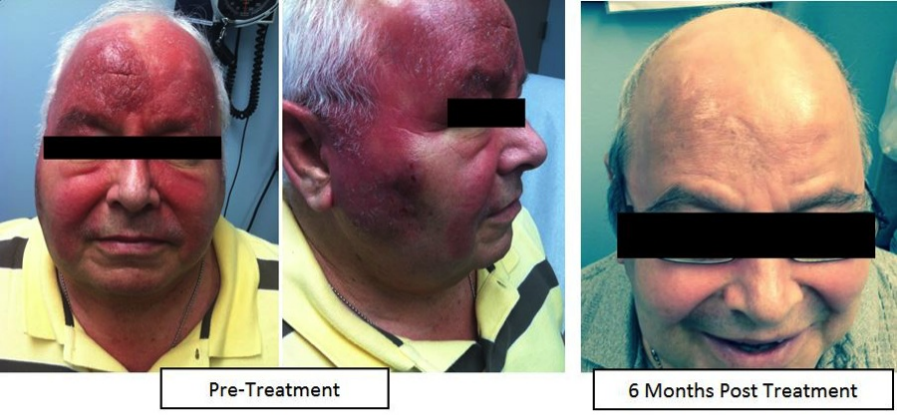
Post-Treatment Response Clinical response at six months post-treatment

At his six-month follow-up, the patient was without evidence of disease, with all areas of involved skin demonstrating a significant improvement with small, residual eczema-like changes; the patient was asymptomatic and overall clinically improved (Figure 7).

## Discussion

Leukemia cutis is defined as a cutaneous infiltration by neoplastic myeloid of lymphoid leukocytes, resulting in clinically identifiable cutaneous lesions [6]. A predominantly B cell infiltration is relatively rare, especially skin-only involvement without clinical involvement to bone or blood. Clinical presentation can be diffuse, and previous radiation series have treated anywhere from the involved site to total body skin irradiation. Targeting the scalp offers several technical challenges, namely attempting to achieve adequate dose coverage in the sub-centimeter region of the epidermis and dermis--an area of electronic disequilibrium--while adequately sparing deeper brain parenchyma and normal tissue structures. Historically, patients with superficial skin and scalp involvement were treated with total scalp irradiation using matched electron fields to parallel opposed photon fields as described by Akazawa, et al., but these techniques suffered from dose inhomogeneity [7]. Furthermore, electron scatter from multiple electron beams along an oblique surface can be difficult to account for dosimetrically. Non-coplanar split field volumetric modulated arc therapy provides a novel approach, leading to optimal dose distribution and conformality. Due to the involvement throughout the scalp, face, chin, neck, and shoulders in our patient, this technique allowed for intensity modulation throughout these irregular anatomical body contours, allowing us to achieve a conformal, superficial dose distribution sculpted around the patient in a ‘bust-like’ distribution. 

## Conclusions

In this case report, we discuss the exceptional response to treatment of a uniquely challenging case of leukemia cutis. The empiric rationale for the decided treatment modality and technique was described in order to further inform the scientific community in the future treatment of these challenging cases.
